# Near-Complete Genome Sequence of GI-17 Lineage Infectious Bronchitis Virus, Circulating in Iowa

**DOI:** 10.1128/MRA.01406-20

**Published:** 2021-05-20

**Authors:** Amro Hashish, Yuko Sato, Ganwu Li, Ying Zheng, Phillip C. Gauger, Mohamed El-Gazzar

**Affiliations:** a Department of Veterinary Diagnostic and Production Animal Medicine, College of Veterinary Medicine, Iowa State University, Ames, Iowa, USA; b National Laboratory for Veterinary Quality Control on Poultry Production, Animal Health Research Institute, Agriculture Research Center, Giza, Egypt; KU Leuven

## Abstract

Avian infectious bronchitis virus (AvIBV) is the causative agent of a highly contagious respiratory disease in chickens which results in significant economic losses in the poultry industry. Here, we report a near-complete genome sequence of the strain, designated IA1162/2020, identified in tracheal swabs from chickens in Iowa in 2020.

## ANNOUNCEMENT

Avian infectious bronchitis virus (AvIBV) belongs to the genus *Gammacoronavirus*, family *Coronaviridae*, subfamily *Coronavirinae*, and order *Nidovirales* (1). AvIBV is characterized by high genetic diversity and the continuous emergence of new variant strains (2). In 2011, a nephropathogenic strain of AvIBV, Delmarva/1639 (DMV/1639), from genotype I lineage 17 (GI-17) was reported (3). Despite the recent wide distribution of this strain in the United States and the availability of multiple S1 gene sequences, whole-genome sequences of this strain are limited. In this study, we report the near-complete genome sequence of a new GI-17 strain, designated IA1162/2020, related to the DMV/1639 strain.

Two pooled tracheal swabs were collected from 10-day-old layer chicks in Iowa with mild respiratory signs and submitted to the Iowa State University Veterinary Diagnostic Laboratory (ISU-VDL) in January 2020. The samples tested positive by reverse transcription-quantitative PCR (qRT-PCR) (4) with a threshold cycle (*C_T_*) value of 18. Total nucleic acid was reextracted from the tracheal swabs using the MagMAX pathogen RNA/DNA kit (Thermo Fisher Scientific, Massachusetts) with a KingFisher 96 instrument (Thermo Fisher Scientific) (5). Double-stranded cDNA was synthesized using the NextFlex rapid transcriptome sequencing (RNA-seq) kit (Bioo Scientific Corp., Texas). The sequencing library was prepared using the Nextera XT DNA library preparation kit (Illumina, California) with dual indexing. The pooled libraries were sequenced on an Illumina MiSeq platform using the 300-cycle v2 reagent kit (Illumina). The raw reads were analyzed and preprocessed using Trimmomatic v0.36 (6). The cleaned reads were classified using Kraken v0.10.5-beta (7) with the standard database. Unclassified reads were classified using Kaiju v1.6.2 (8), and KronaTools v2.6 (9) was used to generate the interactive html charts for the hierarchical classification results. Out of a total of 603,724 reads generated for this sample, 8,214 raw reads were identified as IBV. AvIBV reads were extracted from the classification results for *de novo* assembly using ABySS v1.3.9 (10). The resulting contigs were manually refined and curated to remove contaminated (nonviral) contigs and to trim chimeric (misassembled) contigs in the SeqMan Pro DNASTAR Lasergene 11 core suite. Finally, a near-complete sequence of AvIBV IA1162/2020 (99.9% genome coverage based on the GA9977/2019 reference genome [GenBank accession number MK878536.1]) was identified with a genome length of 27,486 nucleotides, excluding the poly(A) tail, with a GC content of 38%.

Twelve open reading frames (ORFs) were predicted (Table 1) using the ORFfinder program (https://www.ncbi.nlm.nih.gov/orffinder/), resulting in the following genome organization: 5′-1a-1ab-S-3a-3b-E-M-4b-4c-5a-5b-N-3′. This genome (IA1162/2020) is missing ORF 6b, which was supposed to be between the nucleocapsid gene (N) and the 3′ untranscribed region (UTR). ORF 6b (74 amino acids [aa]) is thought to be an apoptosis inducer (11) and accelerated the replication of murine coronavirus (CoV) *in vitro* (12).

**TABLE 1:** Open reading frames, proposed function, nucleotide length and amino acid size of the DMV/1639-like IBV strain (IA1162/2020 strain)

Open reading frame	Proposed function	Nucleotide location		Nucleotide length (bp)	No. of amino acids
		From	To		
1a	Nonstructural polyprotein	522	12383	11,862	3,953
1ab	Nonstructural polyprotein	522^*a*^	20416	19,895	6,631
Spike	Structural protein	20367	23867	3,501	1,166
3a	Accessory protein	23867	24040	174	57
3b	Accessory protein	24040	24234	195	64
Envelope “3c”	Structural protein	24215	24538	324	107
Membrane	Structural protein	24519	25187	669	222
4b	Accessory protein	25188	25472	285	94
4c	Accessory protein	25393	25563	171	56
5a	Accessory protein	25547	25744	198	65
5b	Accessory protein	25741	25989	249	82
Nucleocapsid	Structural protein	25932	27161	1,230	409

a A −1 frameshift for polyprotein 1ab occurs at position 12352.

**FIG 1:**
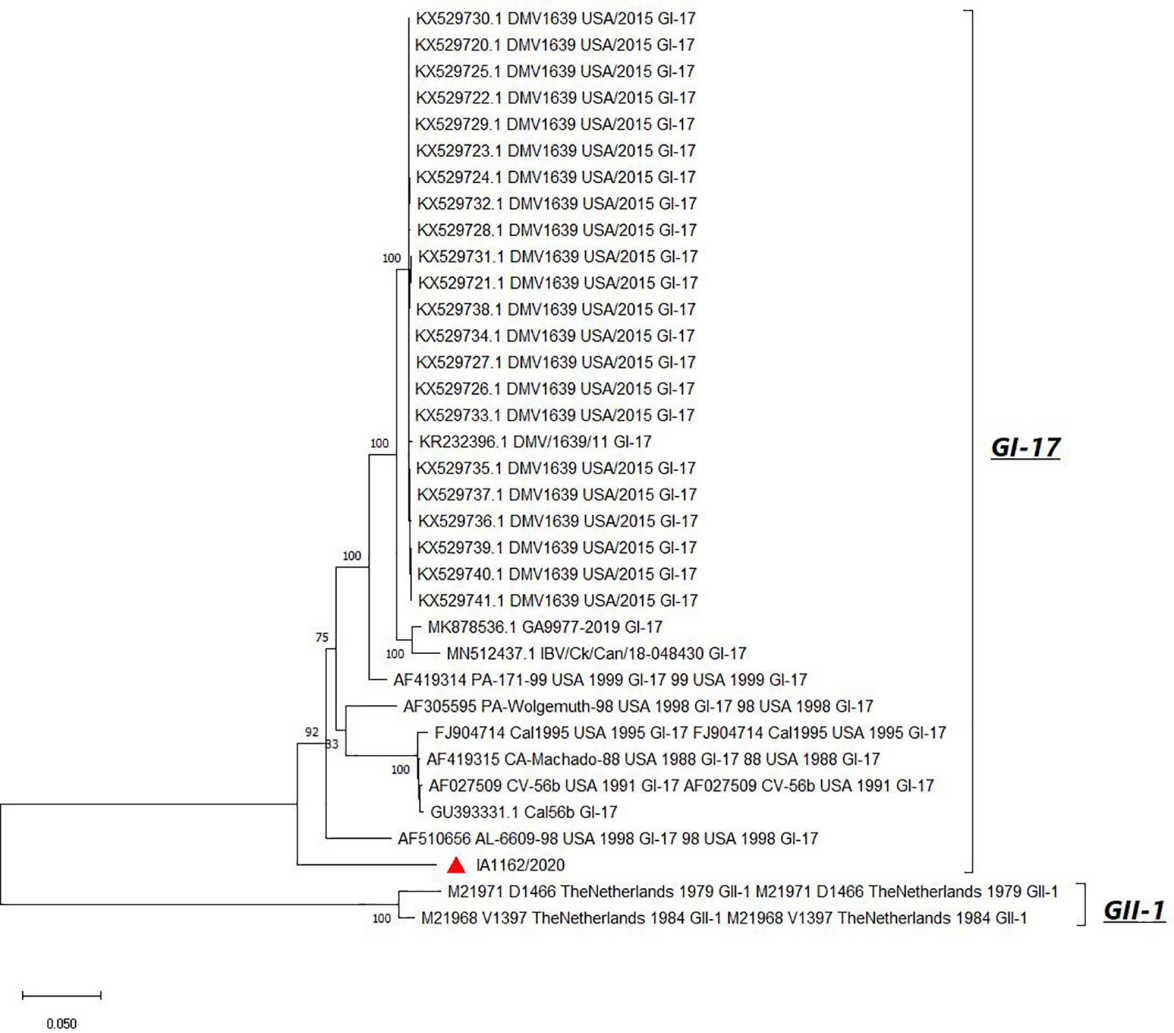
Phylogenetic tree generated from the complete S1 gene sequences of IA1162/2020 and other avian infectious bronchitis virus GI-17 strains. The analysis was done with the neighbor-joining method, Kimura 2 parameters (15), and MEGA X (16). Numbers at the nodes correspond to bootstrap values (1,000 replications). Bootstrap values below 70 were removed. The IA1162/2020 strain is marked with a red triangle. All ambiguous positions were removed for each sequence pair (pairwise deletion option). There were a total of 1,773 positions in the final data set. This analysis involved 35 nucleotide sequences. Sequences from the GII-1 lineage are included as an outgroup.

Full-length genome sequence BLAST comparison of IA1162/2020 showed the highest nucleotide similarity (94.78%) to Georgia strain GA9977/2019 (GenBank accession number MK878536.1) belonging to lineage GI-17 (13). The sequence of the full S1 of IA1162/2020 clustered within GI-17 (14); however, it formed a separate clade away from other GI-17 strains (Fig. 1).

This near-complete genome will be useful for developing new diagnostic assays and new and alternative vaccination strategies and for better understanding the evolution of AvIBV.

### Data availability.

The near-complete genome sequence of the IA1162/2020 AvIBV DMV/1639 strain has been deposited in GenBank under the accession number MW024789. The raw data were deposited under SRA number SRR12575717, BioSample number SAMN15963238, and BioProject number PRJNA660897.
